# Lived Experiences of Women with Substance Use Disorders Regarding Their Motivations and Barriers to Seeking Treatment

**DOI:** 10.1192/j.eurpsy.2026.10205

**Published:** 2026-07-31

**Authors:** E. Albal, G. Sahin-Bayindir, T. Comez-Ikican, I. Alniak, M. Erkiran

**Affiliations:** 1 Adult Detoxification Center, Bakirkoy Prof. Mazhar Osman Mental Health & Diseases Hospital; 2 Mental Health & Psychiatric Nursing, Istanbul University - Cerrahpaşa, Florence Nightingale Faculty of Nursing; 3 Mental Health & Psychiatric Nursing, Istanbul University Faculty of Nursing; 4 Department of Psychiatry, Bakirkoy Prof. Mazhar Osman Mental Health & Diseases Hospital, Istanbul, Türkiye

## Abstract

**Introduction:**

Women with substance use disorders (SUD) face distinct gender-based challenges in accessing and engaging with treatment, including stigma, caregiving responsibilities, and inadequate institutional support. Despite increasing rates of substance use among women, their treatment needs remain under-addressed in both clinical practice and research.

**Objectives:**

This study explored the lived experiences of women with SUD regarding their motivations and barriers to seeking and maintaining treatment in a psychiatric inpatient setting in Türkiye.

**Methods:**

A descriptive phenomenological approach was used. In-depth, semi-structured interviews were conducted with 27 women diagnosed with SUD who had completed detoxification and were receiving inpatient care. Data were analyzed using Colaizzi’s method, supported by MAXQDA 24.0.

**Results:**

Four core themes emerged: (1) Motivation to initiate treatment—awareness of harm, maternal responsibilities, exposure to recovery models, and emotional support; (2) Motivation to maintain treatment—positive clinical relationships, reduced craving, regained self-confidence, structured routines, and hope for the future; (3) Barriers to initiating treatment—fear of stigma, low self-efficacy, social isolation, and substance-using peers; (4) Barriers to maintaining treatment—withdrawal symptoms, emotional distress, and disruptive behaviors within the treatment environment.

**Conclusions:**

Motivations and barriers to treatment among women with SUD reflect an interplay of psychological, social, and structural factors. Addressing these multidimensional needs is essential for sustained recovery. Gender-responsive, trauma-informed treatment environments that enhance therapeutic alliance, peer cohesion, and systemic accessibility are vital to improving treatment outcomes for women with SUD.

**Disclosure of Interest:**

None Declared

